# Maintenance and decline of physical activity during adolescence: insights from a qualitative study

**DOI:** 10.1186/1479-5868-8-117

**Published:** 2011-10-21

**Authors:** Mathieu Bélanger, Michelle Casey, Marc Cormier, Annie Laflamme Filion, Geneviève Martin, Stéphanie Aubut, Philipe Chouinard, Simon-Pierre Savoie, Jacinthe Beauchamp

**Affiliations:** 1 Department of family medicine, Université de Sherbrooke, Sherbrooke, Canada; 2 Dieppe Family Medicine Unit, Dieppe, Canada; 3 Centre de formation médicale du Nouveau-Brunswick, Moncton, Canada; 4 Research Centre, Vitalité Health Network, Moncton, Canada

**Keywords:** qualitative, maintenance of physical activity, decline of physical activity, adolescence

## Abstract

**Purpose:**

Better knowledge on why some individuals succeed in maintaining participation in physical activity throughout adolescence is needed to guide the development of effective interventions to increase and then maintain physical activity levels. Despite allowing an in-depth understanding, qualitative designs have infrequently been used to study physical activity maintenance. We explored factors contributing to the maintenance and the decline of physical activity during adolescence.

**Methods:**

Questionnaires were administered to 515 grade 10-12 students. The Physical Activity Questionnaire for Adolescents was used to determine physical activity level at the end of adolescence. An adapted version of this questionnaire was used to estimate physical activity in early adolescence. Among both genders, we identified participants who maintained a high level of physical activity since grade 7 and some whose activity level declined. For each category, groups of 10 students were randomly selected to take part in focus group discussions.

**Results:**

Seven focus groups with 5 to 8 participants in each were held. Both maintainers and decliners associated physical activity with positive health outcomes. Maintenance of physical activity was associated with supportive social environments and heightened feelings of competence and attractiveness. A decline in physical activity was associated with negative social validation, poor social support and barriers related to access.

**Conclusions:**

Although maintainers and decliners associate physical activity with similar themes, the experiences of both groups differ substantially with regards to those themes. Taking both perspectives in consideration could help improve interventions to increase and maintain physical activity levels of adolescents.

## Introduction

Physical inactivity is the most prevalent preventable risk factor for chronic disease and mortality in Canada [1]. Although the benefits of participation in physical activity (PA) are widely recognized, [2] approximately 95% of Canadian adolescents do not engage in PA to the extent recommended [3]. Moreover, adolescence is generally characterized by marked declines in PA, [4] and adolescents who have low levels of PA are likely to remain insufficiently physically active as they become adults [5]. Studies nevertheless suggest that some individuals succeed in increasing their levels of PA or in maintaining a high level of involvement in the behavior during adolescence [6,7]. Interventions to encourage the maintenance of high levels of PA throughout adolescence may be improved by taking into account how those who maintain high levels of PA differ from those with low or declining PA levels.

Systematic reviews have identified correlates that are robustly associated with PA. For example, gender, socioeconomic status, psychological characteristics, friends, family, and physical environment all relate to different activity levels [8-10]. However, an in-depth understanding of why some adolescents discontinue or maintain PA participation in the presence of these correlates is lacking. Whereas qualitative studies could help understand how various factors interact to lead to various behavioral patterns, only a handful of studies have qualitatively explored reasons for PA declines during adolescence. These show that common reasons for taking part in PA include enjoyment, social interactions and weight management, whereas lacking confidence and ability are often cited as barriers to participation [11-14]. These elements are all components of the Theory of Reasoned Action and Planned Behavior (TPB). The TPB stems from the concept that behaviors are modeled through the intentions of an individual, and that these intentions are modified by three main elements interacting with each other: attitudes towards the behavior, subjective norms, and perceived behavioral control. The theory suggests that if these three components are viewed in a positive way in relation to a specific task, the intention of completing the task will be stronger. Attitudes towards the behavior can be defined as positive or negative feelings regarding physical activity, in other words the perceived benefits and/or consequences associated with being physically active. Perceived pressures from parents, teachers and the media reflect subjective norms towards physical activity, and perceived behavioral control is represented by one's perceived ability to accomplish a task despite internal and external barriers [15].

Previous qualitative investigations focused on a cross-sectional comparison of physically active and inactive individuals and did not consider differences among elements that contributed to maintaining or declining PA levels. While there is interest in understanding how physically active and inactive individuals differ, there is additional value in studying differences between PA maintainers and decliners since PA interventions should not only aim for an increase in PA participation, but also for the maintenance of elevated PA level. The objective of this study is therefore to develop a better understanding of factors associated with the maintenance and the decline of PA during adolescence. More specifically, the aim is to use qualitative research methods to explore how adolescents who maintained high levels of physical activity differ from those who went from being very physically active to now taking part in little physical activity in relation to their PA-related attitudes, subjective norms and perceived behavioral control. We use the TPB as a framework for this study as literature shows that it can explain about half of the variance in intentions to increase PA [16] and that the perceived behavioral control component of the theory was significantly predictive of exercise maintenance among adults [17].

## Methods

### Participants

Participants were recruited from two French-speaking secondary schools in New Brunswick, Canada during the 2008-2009 and 2009-2010 school years. The schools were conveniently selected based on their principal's interest in the study and relative proximity from the study center. 1255 students were in grades 10, 11 or 12 and ranged from 15 to 18 years old. This study received ethical approbation from the Vitalité Health Network institutional review board.

### Data collection

This study included two phases. In phase one, students were invited to complete a questionnaire. This questionnaire was used to determine participant categories (maintenance or decline of PA). Phase two consisted of focus groups with participants from each category. Focus groups were chosen because they have the advantage of collecting quickly a broad range of ideas on a topic [18]. They also provoke social interactions which can help reveal how a group represents issues with built-in checks and balances by participants with different points of view [18,19].

#### Physical activity categories

Teachers in all grade 10, 11 and 12 classes read a description of the study and directions aloud and distributed questionnaires to students assenting to participate in the study. Once completed, questionnaires were placed and sealed in opaque envelopes before being sent to the schools' secretary office for pick-up by study personnel. Current PA level (late adolescence) was measured with the Physical Activity Questionnaire for Adolescents (PAQ-A). The PAQ-A is an eight-item questionnaire which assesses usual PA at various times of the week and weekend days [20,21]. It has moderate to high reliability (test-retest *r *= 0.75-0.82) and correlates moderately with accelerometers (*r *= 0.39) [20,21]. Past PA was measured with the item: "Which of the following best represents the physical activity level you had in grade 7", followed by five descriptions: 1) All or most of my free time was dedicated to do things that demanded little physical activity; 2) I sometimes (1-2 times per week) did physical activities during my free time; 3) I often (3-4 per week) did physical activity during my free time; 4) I quite often (5-6 times per week) did physical activity during my free time; 5) I very often (7 or more times per week) did physical activity during my free time. We pilot tested this question by administering it to 8 adolescents and interviewing them individually to verify clarity.

Participants categorized as "low level of PA" during grade 7 (response options1 or 2, i.e.: report of fewer than 3-4 PA sessions per week) were excluded from this study. Participants with reports of being physically active 3-4 or more times per week in grade 7 were categorized as previously physically active and were categorized further as "maintainers" or "decliners" according to their score on the PAQ-A (≥3 and < 3, respectively). We excluded participants for which this categorization did not converge with their own perception, measured with the item: "When you were in grade 7, would you say that you were: a lot more physically active than this year; a little more physically active than this year; at a similar physical activity level as this year; a little less physically active than this year; a lot less physically active than this year."

#### Focus groups

Our plan was to hold two gender-specific focus groups with each of PA maintainers and decliners separately (total of 8 groups). The division of groups according to gender and PA pattern aimed at making participants as comfortable as possible to share their thoughts. For each group, 10 randomly selected adolescents were invited to participate. A semi-structured discussion guide was developed by reviewing relevant literature and by drawing on the researchers' experience. The guide consisted of four main open-ended questions with examples of sub-questions to probe further as necessary. The discussion guide was developed with the aim of understanding what were perceived as factors associated with the maintenance or decline of PA and enabled all domains of the TPB to be represented [22]. The questions were nevertheless vague enough to allow flexibility in the direction participants would take the discussion. Specifically, questions and probes targeted attitudes regarding PA as well as individuals, contexts and barriers influencing PA behavior and intentions. The main questions used were: "What do you like about physical activity?", "What motivates you to do physical activity?", "Who do you do physical activity with?" and "Over the past few years, what contributed to you continuing or not to do physical activity?" Women moderated girl-groups and men moderate boy-groups to facilitate discussions. All discussion moderators were trained to initiate the discussion by explaining the purpose and structure of the meeting and encouraged participation from all participants. The 50-60 minutes discussions were audio-recorded. In addition, the researchers took field notes which were used as a starting point for analyses. Citations were transcribed in French and were translated in English at the time of manuscript writing.

### Data analysis

Three research team members independently conducted a thematic analysis of the data. Thematic analysis is a simple method and the basis of most qualitative data analyses [23]. It allows for representation of the whole of a data corpus. It also has the advantages of generating unanticipated insights as well as producing analyses suited to informing policy or intervention development [24]. Thereafter, four research team members (including the three analysts) discussed the themes identified in the analysis. Together they agreed on common themes and used them to code elements of the discussions. The list of themes was then further refined by grouping themes that were strongly interconnected and by referring to the TPB components. The final coded data were reviewed and validated by two other team researchers who had facilitated focus group discussions.

## Results

**Figure 1:**
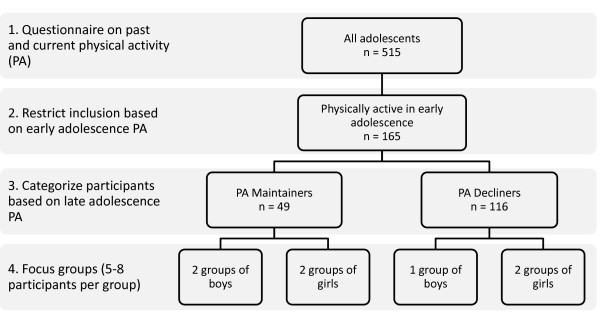
**Summary of study procedures**.

Of the 1255 students in grades 10-12, 515 (41%) completed the questionnaire. Of those, 126 were not analyzed because: gender was not identified (11), questionnaire was filled with implausible responses (59), or participants were in grade 9 or lower (56). A further 224 questionnaires were not retained because respondents were considered to have low levels of PA in grade 7 (157) or had discrepancies between their perceived and measured change in PA level (67). Of the remaining participants, 23 boys and 26 girls were categorized as maintainers and 35 boys and 81 girls as decliners (Figure 1). Two focus groups were held with each of maintainer-boys, maintainer-girls, and decliner-girls, whereas only one group was held with decliner-boys. Group size ranged from 5 to 8 participants. There were no meaningful or statistically significant differences in age or current physical activity level between participants in the focus groups and those who were invited but did not participate in the focus groups.

Five general themes emerged from the analysis. Although there were few differences based on gender in the identification of themes, marked differences were noted in terms of the importance of some of the themes for PA maintainers and decliners. The five themes and their particularities related to either gender or PA categories are described below. The results are summarized in Table 1.

**Table 1:** Factors reported to be associated with physical activity maintenance among adolescents who maintained or declined involvement in physical activity

Theory of Planned Behavior components	Themes		Adolescents who maintain PA	Adolescents who decline PA
Attitudes	Benefits/Consequences	Mental health	+	+
		Acute disease prevention	+	
		Better performance	+	
		Social interactions	+	-
		Body image	+	
Subjective norms	Social support and pressure	Parental support	+	-
		Role modeling	+	
		Social environment pressure		-
		Social validation	+	-
	Media	Influence from celebrities	+	
Perceived behavioural control	Perceived competence	Feeling of competence	+	-
	Access	Cost, time, availability		-
		Parental support	+	-

PA = physical activity; + denotes factors reported to be positively associated with the maintenance of physical activity throughout adolescence; - denotes factors reported to be negatively associated with the maintenance of physical activity during adolescence.

### Theme 1: Benefits/Consequences

A variety of physical and psychological health benefits were cited as motives for being physically active. Participants of both PA categories positively described a link between PA and mental health in very specific terms, for example "feeling good mentally" and "relieving stress". One participant suspected that if she had not been active on a daily basis, she would have suffered from depression following a breakup in a relationship. Although mental health benefits were reported by most participants, only maintainers associated PA with other health benefits. In particular, maintainers suggested that being physically active provides "more energy" and that physically active individuals are not as affected by acute diseases such as colds and influenza as those who tend to be sedentary.

People who are often sick, are the ones that don't do sports. (Maintainer boy) Maintainers indicated that another motive for taking part in PA was to be able to perform well in the sports that they practice. This implies that some of the maintainers take part in at least two types of PA: one that they enjoy and one that provides them the fitness required to enjoy the activity. One maintainer boy summed this up by saying:

When you're fit, it's more fun to exercise because you perform better.

An important motivator for maintaining participation in PA was also the heighten gratification in relation to having a lean and muscular body figure. The importance of actual and desired appearance as a motive to be physically active emerged more strongly among the maintainer-boys than any of the other groups. Participants mentioned how they would get involved in a sport or continue doing a PA to preserve a body image of which they were proud. Physical activity was seen as a tool to develop muscle mass in order to become more sexually attractive as this boy explained:

You want to attract girls, have a 6-pack, have more arms. You try to push more, so you train more to have more muscle... so you train more seriously.

Maintainers also appeared to take part in physical activity because they enjoyed the behavior. One of the most important elements of enjoyment was related to the social aspects of team sports and physical activities practiced with peers:

With team sports, the chemistry in the team, that is what's fun. (Maintainer boy)

My best friend dances with me and it's way more motivating cause then you know you won't be alone when you need to go somewhere, and you tell yourself it's going to be real fun. (Maintainer girl)

### Theme 2: Social support and pressure

Participants reported how their social environment affects their involvement in PA. Family, parents in particular, appeared to strongly influence the PA patterns of participants. Among the maintainers, it was often reported that parental support facilitated being physically active. Parents provided moral support (ex: attending sporting events, giving encouragement) and therefore contributed to the creation of subjective norms, but they also brought material support (ex: driving to the practices, purchasing equipment) which contributed to the perception of behavioral control.

Had I felt I bothered my parents because I needed drives everywhere, I obviously wouldn't have stayed in the team. But they like it. You really can't do it without your parents. (Maintainer girl)

Maintainers also communicated how role modeling from parents and other family members influences how active they are.

I grew up with two brothers and they were active, so it had an influence on me. (Maintainer girl)

Decliners also recognized the importance of parental support for being physically active. However, their experience with parental support was more negative as this girl testified:

My parents never come watch my games, it's kind of sad. I'd like them to come. (Decliner girl)

They described how not being supported by parents could be conducive to adopting a physically inactive lifestyle.

I was just tired of asking them (parents) for drives so I just quit. (Decliner girl) Peers also had an influence on participants' PA. However, participants reported more frequently developing friendships with team members and others taking part in the same activity rather than following friends into the activities (e.g.: "You meet new people, it's fun", Maintainer boy). The influence of peers often appeared to come in the form of pressure to be active or to perform. Both groups of PA category reported examples where they felt pressure from friends, coaches, or family members to be physically active. These reports were more frequently heard among girls than boys. For the decliners, the social pressure to be physically active was difficult to manage and was a precursor of their physical activity decline:

I was playing with the soccer team last year; I didn't go this year just because it was too much pressure. It's like I was feeling like I didn't play my best because I had too much pressure on me, and I wasn't playing like I know I can play. (Decliner girl)

One girl explained that the social environment surrounding team sports put too much importance on performance and consequently eliminated fun from organized physical activities:

Everybody has the right to play in house league, but it's always the same that played... lots of people didn't show up to games anymore. All the coach wanted was to win... everybody's allowed to have fun.... (Decliner girl)

In contrast, pressure was perceived as a positive source of motivation among the maintainers. Two girls in this group explained this dichotomy:

If you have a coach you want to continue (to play) if he encourages you.

There are some (coaches) that discourage you and it doesn't help. There are some that just don't make you play and it makes you play bad.

### Theme 3: Media

For the maintainers, media also appeared to be an important source of influence. For many, watching professional athletes and Olympians on television was inspirational and motivating. As one maintainer-girl put it:

When I was a kid and watching sports on television, I would tell myself, I want to be that girl.

Some maintainers also reported that sports shown on television and in movies were precursor to their involvement in PA (e.g.: "I watched Karate Kid as a kid and then I was like, I want to do that. So I registered in Taekwondo", Maintainer girl). Besides arousing their own interest, maintainers suspected that when sports are largely distributed in mainstream media, it has a similar effect across the general population.

With the Olympics right now, I'm sure that after the Olympics you'll see more people go curling, skating, and do other sports. (Maintainer boy)

### Theme 4: Perceived competence

Physical activity was both a cause and an outcome of feelings of competence. For example, some maintainers reported experiencing a sense of accomplishment and pride following a successful sporting performance which in turn encouraged them to take part in more PA. Among decliners, the relationship between PA and feelings of competence was a barrier to PA. They appeared to compare themselves to peers and negatively evaluate their own skill levels:

I tried figure skating when I was younger, and I quit because my friend was further in the sport..." (Decliner girl)

In some cases, decliners so devalued their skills that it led them to discontinue participation in sports despite their enjoyment of the activity. Decliners provided several examples whereby they opted to remove themselves from situations where their performance risked being judged unfavorably (e.g.: "I quit volley ball even though I love it just because people thought I wasn't good", Decliner girl). Such interruptions of activity appeared to be made in the interest of preserving an image of competency. Although maintainers did not share examples of such negative experiences, some discussed the need to be physically active for intrinsic reasons and to ignore negative external evaluations or comparison with others. One maintainer-girl put it this way:

If you have a low self-esteem (...) and you tell yourself you're not good [at a sport] (...) it will complicate things, and you'll have less fun, and it will push you to do less exercise, so you'll stay home.

### Theme 5: Access

Among the decliners, difficulties getting access to activities they liked were frequently reported. In some instances, access limitations were related to specific activities not being available past a certain age. Participants explained that, as they get older, fewer people register for sports, meaning that there are fewer teams which in turn decreases the opportunities for some to find a team with which to play. According to decliners, towards the end of adolescence, access to competitive sports becomes reserved to the most athletically inclined. Access is reduced further for those living in rural areas given that organized activities tend to take place in urban centers.

Another access related issue reported by decliners was cost associated with activities. Cost of registration and equipment as well as travel expenses required for competitive sports (i.e. hotels and restaurants) limits adolescents' access to some physical activities (e.g.: "the cost of registration goes up every year... it's pretty bad", Decliner girl).

Time was also a concern for some decliners who reportedly discontinued participation in PA because they did not like the time frames (early morning and weekend nights) associated with organized physical activities. For them, these times generally interfered with their opportunities to take part in social events and other activities. These other activities become increasingly important for some adolescents as they get older. Increase in school work and part time employment also impose time constraints.

At our age, PA drops because there are new priorities like school and work which are more important. (Decliner boy)

Time constraints did not appear to be an issue among the maintainers. Rather, some maintainers talked about the organizational skills they developed in relation to having to manage busy agendas.

You have to organize your time because you have school and sports. For me, it helped me to get organized a lot. (Maintainer girl)

## Discussion

This study explored factors contributing to the maintenance or decline of PA levels during adolescence. Although similar themes emerged across the different groups, PA decliners and maintainers had varying views or experiences regarding some of those themes. All components of the TPB were represented. Attitudes towards physical activity, the first component of the TPB, were expressed by participants in the forms of benefits or consequences associated with the behavior. More specifically, PA maintainers described health benefits, enjoyment, body image, social interactions and better performance as motives to engage in PA, suggesting that positive attitudes towards PA increased intent and ultimately led to the maintenance of the behavior. Both maintainers and decliners nevertheless identified health benefits as motives for being physically active. However, in contrast with the frequently reported ability of PA to prevent numerous chronic diseases [25], participants in this study almost exclusively referred to immediate health benefits of PA. This is consistent with other studies suggesting that prevention against chronic diseases does not appear to contribute highly to the motivation to take part in a physically active lifestyle among adolescents [26-28]. These results indicate that interventions promoting PA among adolescents should accentuate the short-term health benefits of the behavior. Other studies have also suggested putting greater emphasis on the wide range of health benefits associated with PA [27]. Adolescents who maintained PA in this study perceived that this behavior provided them with a sense of competence and contributed positively to their body-image. Previous reports have also shown that highly physically active adolescents value muscularity [29] and feel empowered by the social recognition associated with their sport performances [28]. Analogously, it has been suggested that PA could be promoted as a measure for adolescents to obtain positive social feedback and improve body shape [30].

Although involvement in PA often results in increased opportunities for social interactions, PA decliners in this study suggested that these interactions may translate into negative experiences which may actually lead to a discontinuation of PA. Given that negative experiences were often reported to occur in the context of performance based physical activities, future interventions should consider promoting activities that are not made up of overly competitive aspects. As demonstrated recently, such activities also often have the advantage of being more likely to be maintained throughout adolescence [31].

Some themes appeared to contain elements relating to more than one component of the TPB. This suggests a potential interaction between components of the theory to influence intentions and PA. An example of this relates to the themes under which maintainers and decliners identified the influence of their family and peers as a factor contributing to each of their respective PA patterns. It was noted not only that parents' perceptions of PA is important, but also that their support in regards to resources played a role in shaping the PA patterns of participants. Together, the parents' positive perceptions of PA (subjective norms) and their willingness to facilitate transportation (perceived behavioral control) were associated with PA participation among maintainers, whereas the opposite was observed in the group of decliners. A recent study also showed that adolescents with little peer support for PA and physically inactive parents tend to be the least physically active [32]. These contrasting effects likely relate to variations in the social environment of maintainers and decliners. This is in accordance with the TPB which suggests that the social or environmental pressures that surround a behavior (subjective norms) can be positive or negative by increasing or decreasing the intention to participate and thereby influencing the behavior [15].

The difference in effect of peer influence between the two groups may also relate to variations in skill levels of adolescents. Adolescents who believe their peers regard them as athletically competent have been found to exhibit more positive feelings towards PA [33]. In comparison, adolescents who perceive they lag behind their peers in terms of skill level for sports may avoid exposing themselves to PA in order to preserve pride as it was shown that athletic competence is an important social status determinant for youth [34]. This would be consistent with results showing that children who perform better in sports are more likely to sustain PA [35,36]. Our findings show that negatively perceived competence presented a barrier to PA, which supports the perceived behavioral control component of the TPB. Internal barriers, such as negatively perceived competences, can therefore decrease intents to be physically active, thus indirectly affecting behavior through intention. Perceived competences of participants were nevertheless also connected to the subjective norms. Participants in this study frequently discussed about perceived competence concurrently with elements of social validation (i.e.: if you are competent, your peers will judge you favorably). This therefore represents another example whereby two components of the TPB (perceived behavioral control and subjective norms) interacted with each other to ultimately influence PA.

Besides some internal barriers, external barriers were also noted to be associated with PA decline. Lack of time, cost, transportation, and whether an activity is available were all reported to influence PA. These barriers exemplify the direct influence of the perceived behavioral control component of the TPB on PA. For example, even if an individual has the intention to participate in PA, a time constraint, lack of transportation or a high cost of participation can inhibit the behavior from taking place. Throughout the group discussions, participants appeared to mainly associate PA with leisure and talked almost exclusively in terms of sports, teams, or competition. Participants generally discussed little about other aspects of PA (occupational, transport, and domestic activities) despite having been provided with a general definition of PA at the beginning of the discussion. If confirmed in future studies, such results could justify interventions to inform the population of all means through which recommended PA levels can be attained. In light of our findings, the media could play an important role in disseminating such information. The promotion of additional methods of attaining an active lifestyle could contribute to normalizing PA by increasing a sense of accessibility and decreasing the perception that PA is reserved for the most affluent and athletically inclined. It would also correspond better with the fact that the majority of activities contributing to the attainment of physical activity guidelines are not exercises or sports [37].

Strengths of this study include that the focus groups were separated by gender and by PA pattern of adolescents and that participants came from the general population of students in two schools. Although it is unlikely that participants were misclassified as maintainers or decliners, it is possible that those who participated in the focus groups differed from the non-participants who were eligible. It is also possible that participants overestimated their current and past PA level. Long-term recall of PA level imposes some limits and the question used to estimate past PA level of participants has not been formally validated. However, pilot testing of the instrument suggested that adolescents understand the question perfectly. It must nevertheless be reminded that assessing the PA behavior of participants was not the goal of this study and that questionnaires are considered suitable for categorizing individuals based on their activity level [38]. Although focus groups are a useful method to highlight a variety of views as well as contradictions and tensions between participants' opinions, limitations include the possibility that mainly socially acceptable points of view were shared and discussed. As well, it is possible that some participants dominated the conversation and imposed their views. Participants may also have retained some of their less socially desirable opinions because of the presence of the discussion moderators. Finally, uncontrollable logistic issues, including a snow storm on one day of data collection and a failure to announce the study in some classes, mean that only one focus group was held with decliner-boys.

## Conclusion

This may be the first qualitative investigation to compare individuals who maintained and individuals who decreased their level of participation in PA during adolescence. Findings indicate that the TPB is useful to present factors associated with these two patterns of involvement in PA. Attitudes, subjective norms, and perceived behavioral control all related to the different PA patterns of participants and may therefore serve as a guide for planning interventions aiming at maintaining high levels of participation in PA. We have shown that although maintainers and decliners associate PA with similar themes, the experiences of both groups differ substantially with regards to those themes. Taking both perspectives in consideration could also help improve interventions to increase and maintain PA levels of adolescents. As such, results support interventions that focus on immediate health benefits of PA, including its impact on body image, promotion of non-competitive and individual physical activities, identification of physically active role models, and easing access to PA.

## Competing interests

The authors declare that they have no competing interests.

## Authors' contributions

MB, SA, PC, and SPS conceived the objectives of the study and designed the protocol. MB, JB, MC, MC, ALF, and GM carried out analyses and interpreted the data. The writing of the manuscript was led by MB, but all authors provided comments and contributed to the manuscript writing. All of the authors reviewed the manuscript critically for important intellectual content and approved this final version.
